# A Network Analysis of Suicidal Cognition as a Central Node Linking Depression and Prospection Bias in University Students

**DOI:** 10.3390/bs16060893

**Published:** 2026-06-02

**Authors:** Lei Xia, Zhuoya Yang, Qianyu Zhang, Yixiao Fu, Zhengzhi Feng, Chunmeng Shi

**Affiliations:** 1 Experimental Research Center for Medical and Psychological Science, School of Medical Psychology, Army Medical University, Chongqing 400038, China; xialei@tmmu.edu.cn; 2 Department of Basic Psychology, School of Medical Psychology, Army Medical University, Chongqing 400038, China; yangzhuoya@tmmu.edu.cn; 3 School of Public Administration, Chongqing University, Chongqing 400044, China; 27206694@alu.cqu.edu.cn; 4 Department of Psychiatry, The First Affiliated Hospital of Chongqing Medical University, Chongqing 400016, China; fuyixiaoresearch@126.com; 5 Department of Medical Psychology, First Affiliated Hospital of Army Medical University, Chongqing 400038, China; 6 State Key Laboratory of Trauma, Burns and Combined Injury, Institute of Rocket Force Medicine, Army Medical University, Chongqing 400038, China

**Keywords:** depression, prospection bias, suicide, network analysis, university students

## Abstract

Prospection bias (PB) plays a crucial role in the development and maintenance of depression. However, the symptom-level mechanisms linking specific depressive symptoms to distinct PB components remain unclear, limiting the precision of targeted interventions. This study employed a network analysis approach to elucidate the intricate symptom-level relationships between depression and PB in a large sample of 1162 university students (453 males and 709 females; mean age = 19.47 years, SD = 1.45). Participants completed self-report assessments of depressive symptoms and prospection bias. The network demonstrated high stability. Central symptoms within the depression–PB framework were identified as “suicidal ideation”, “blurry future imagery”, “lack of energy”, and “catastrophic negative future thinking”. Critically, “suicidal ideation”, “future imaginations of self-harm/suicide”, and “guilt” served as the pivotal bridge symptoms connecting depression and PB. The Network Comparison Test revealed no significant gender differences in network structure. These findings highlight that current suicidal ideation and future-oriented suicidal imagery may serve as key nodes in the network of depressive symptoms and prospection bias. The results may help inform early identification and targeted intervention among at-risk university students.

## 1. Introduction

University students occupy a developmentally critical transition from adolescence to adulthood, a period characterized by heightened stress reactivity and identity consolidation. Meanwhile, they are facing unprecedented challenges, including academic pressure, economic burdens, and social adaptation. Consequently, this population exhibits elevated vulnerability to depression, with previous studies reporting a high prevalence of depressive symptoms among university students (Z. Z. Lin et al., 2025). For example, depressive symptoms have been reported in approximately 38.70% of university students (Z. Z. Lin et al., 2025), while the prevalence among 18–24-year-olds in the general population was estimated at 24.10% (Fu et al., 2022). Depression not only impedes academic performance and leads to social withdrawal, but it can also provoke suicidal ideation and other personal developmental crises, resulting in substantial socioeconomic burdens (Herrman et al., 2022). Elucidating the cognitive mechanisms underlying depression in this population is therefore essential for precision prevention.

The early literature used the term “prospective bias” to describe the mental simulation of future events that is biased toward either positive or negative representations of possible future outcomes (Namaky et al., 2021). Following Yang et al. (2023), the present study conceptualized prospection bias (PB) as a cognitive distortion in prospection driven by the cognitive schema bias in the context of depression, encompassing increased negativity (excessive negative experiences), reduced positivity (lack of positive experiences), and overgeneralization (reduced specificity and vividness; Yang et al., 2023). PB plays a crucial role in depression. Beck et al.’s (1979) cognitive triad posits that negative cognitions regarding the world, the self, and the future are not only central manifestations of depression but also crucial factors contributing to its onset, maintenance, and recurrence (Beck et al., 1979). Recently, the “prospection in depression” model claims that PB is one of the key factors driving the onset of depression (Roepke & Seligman, 2016). The association between PB and depression has been validated across different populations. Studies have indicated that both patients with major depressive disorder (MDD) and dysphoric individuals demonstrate reduced specificity, vividness, and anticipatory pleasure when envisioning positive future events while exhibiting a greater intensity of negative experiences when imagining negative events (Hallford, 2019; Tang et al., 2023). Previous research suggests that suicide-related future imagery is closely associated with suicidal ideation in depressed and formerly suicidal patients (Holmes et al., 2007). Furthermore, longitudinal studies have found that reduced positivity for the future can predict subsequent depressive symptoms and suicidal attempts (Kube et al., 2020). However, previous studies have only explored the associations between certain components of PB and overall levels of depression. It remains unclear how specific PB and depressive symptoms interact to drive the development of depression.

In recent years, prospection training achieved initial effectiveness in improving depression in both subclinical and clinical settings, primarily by increasing specificity and anticipatory pleasure to reduce overall depressive levels (Fischer et al., 2022; Hallford et al., 2022). However, these prospection trainings did not take into account the important distinctions and unique connections between different symptoms. Clarifying the relationship between specific PB and depression symptoms could provide precise targets for future interventions, thereby enhancing the effectiveness of interventions for depression prevention and treatment.

In accordance with network theory, depression may arise from the interactions among various factors rather than from a single underlying cause (Borsboom, 2017). Accurately mapping these interactions is crucial for elucidating the mechanisms of psychopathology and tailoring targeted interventions. Consistent with this perspective, network analysis emerges as a valuable approach for examining the intricate connections between PB and depression. This method models psychopathological structures as interconnected symptom nodes, with edges representing associations between nodes while controlling for other variables within the network (Borsboom & Cramer, 2013). Moreover, it provides meaningful indices to evaluate the significance of nodes, such as the strength centrality index and the bridge centrality index.

The present study leverages psychometric network analysis to construct an integrated symptom network model of depression and PB in university students. Specifically, we aim to (1) identify central symptoms that activate and maintain the depression–PB network, and (2) detect bridge symptoms that transmit activation between depressive and prospection components. By elucidating these mechanistic pathways, we seek to inform targeted prevention strategies for this high-risk population.

## 2. Method

### 2.1. Participants

This study conducted a cross-sectional survey of university students in Chongqing, China. Undergraduate students from five universities were recruited to complete online questionnaires. Recruitment information, including a brief description of the study and a survey link/QR code, was distributed through university or class-based online groups. Participation was voluntary and anonymous. Prior to the assessment, all participants signed an electronic informed consent form. The questionnaire was administered through an online survey platform (https://www.wjx.cn/). All items were set as mandatory, and the questionnaire could not be submitted if any required item was left unanswered. The inclusion criteria were as follows: (1) being an undergraduate student enrolled at one of the five universities; (2) being able to read and understand Chinese; and (3) providing electronic informed consent. The exclusion criteria were as follows: (1) the answer was “No” to the question “Did you read each item carefully and answer truthfully?”; (2) either of the lie detection questions (“I have never had a cold, even if it is only very slight”, “I never tell lies, even if it is only a small lie”) was scored greater than 4; (3) a response time less than the bottom 2.5%, specifically a completion time of less than 188 s. This study was approved by the Ethics Committee of Army Medical University.

A total of 1325 questionnaires were collected. After applying the exclusion criteria, 1162 questionnaires were valid with an effective response rate of 87.70%. Therefore, the 1162 participants were included in the analysis, of whom 709 were female (61.02%). The mean age was 19.47 years (SD = 1.45). The Patient Health Questionnaire 9 scale (PHQ-9; Q. Lin et al., 2021) was used to evaluate depression symptoms. Based on the PHQ-9 severity categories, 617 participants had minimal depressive symptoms (0–4), 357 had mild symptoms (5–9), 131 had moderate symptoms (10–14), 44 had moderately severe symptoms (15–19), and 13 had severe symptoms (20–27) (Table 1).

### 2.2. Measures

The Patient Health Questionnaire 9 scale (PHQ-9; Q. Lin et al., 2021) consists of 9 items and is used to screen and evaluate depression symptoms in the past two weeks using a 4-point rating scale (0 “not at all” to 3 “almost every day”); total scores range from 0 to 27, with higher scores indicating more severe depression symptoms (0–4 = no depression, 5–9 = mild depression, 10–14 = moderate depression, 15–19 = moderately severe depression, 20–27 = severe depression). Studies have shown that the PHQ-9 has good reliability and validity (Richardson et al., 2010). The scale demonstrated good internal consistency in the current study (Cronbach’s α = 0.85).

The Negative Bias in Prospection Scale (NBPS; Yang et al., 2023) consists of 14 items and is used to assess the negative bias in prospection. The scale consists of three factors: increased negativity (excessive negative experiences), reduced positivity (lack of positive experiences), and overgeneralization (reduced specificity and vividness), which is scored on a 5-point rating scale (1 “completely disagree” to 5 “completely agree”). The NBPS is an easy and efficient tool; total scores ranging from 14 to 70, with higher scores indicating higher levels of prospection bias. The NBPS showed good internal consistency in this study, with a Cronbach’s alpha coefficient of the scale of 0.92.

### 2.3. Statistical Analysis

This study used SPSS 26.0 for descriptive analysis, using means and standard deviations to describe depression, and PB scores of university students. The network analyses were performed using R 4.4.1 software.

We used Spearman correlations to assess the correlation between the items of the PHQ-9 and the NBPS, which is relatively robust to skewness and outliers. The enhanced least absolute shrinkage and selection operator (eLASSO) method was applied to construct the network model (Friedman et al., 2008). This method uses a penalty parameter to achieve sparsity and utilizes the extended Bayesian information criterion (EBIC) to determine the optimal set of factors near each node (Foygel & Drton, 2010). The EBIC tuning parameter γ was set to 0.5. This process requires the use of the R-packages “qgraph” and “bootnet”, ultimately resulting in a visual network. The network consists of “nodes” and “edges”, where each symptom is considered a node and the correlation between two symptoms is considered an edge. After each node is connected to several other nodes, the network is automatically constructed, displaying the strength of direct relationships between nodes. The network distribution is such that nodes with more frequent and stronger associations with other nodes are located at the center of the network. In graphs, edges of different colors represent different directions of association (red edges indicate negative correlation, green edges indicate positive correlation), and the strength of association between nodes is represented by the thickness of the lines.

Based on previous research (Borsboom & Cramer, 2013), we used the strength centrality index to represent the importance of individual symptoms within the model. Strength is defined as the sum of the correlations between a given node and all other nodes, with higher values indicating greater centrality within the network. This analysis was conducted using the “qgraph” package in R, and results were reported as standardized scores (z-scores). Additionally, we analyzed the bridging centrality of each node in the network using the “networktools” package in R. Bridging centrality serves as an indicator to evaluate the role of symptoms between two clusters (Jones et al., 2021). It reflects the importance of a node that connects to all nodes in another cluster, with higher values signifying greater importance of that node. Moreover, we utilized the R package mgm to calculate the predictability of each node. Predictability refers to the extent to which the variance of a node can be explained by its neighboring nodes. Predictability can reflect the controllability of the network: when the predictability of a node is high, we can control it via its neighboring nodes; when the predictability of a node is low, we can directly intervene on it or look for other variables outside the network to control it (Haslbeck & Waldorp, 2018).

According to the recommendations of previous studies (Borsboom et al., 2018; Epskamp et al., 2018), we utilized the R package “bootnet” (version 1.4.3) to assess the robustness of the network model, specifically by evaluating the accuracy of edge weights and the stability of centrality indices. We employed a non-parametric bootstrap method to compute 95% confidence intervals (CIs) to evaluate the accuracy of edge weights. Subsequently, we applied a sample-reduction bootstrap method to calculate the correlation stability (CS) to assess the stability of centrality indices (in this study, the strength). The CS value indicates the maximum proportion of the sample that can be removed; generally, the CS coefficient should not be lower than 0.25, with a preference for values exceeding 0.5. Finally, to examine whether there were significant differences in node strength and edge weights, we employed a non-parametric bootstrap difference test, repeating the sampling process 1000 times.

Using the network comparison test in the R package “NetworkComparisonTest” (NCT), we employed permutation testing to examine the structural differences between two estimated models (Borkulo et al., 2022). This method compares network structures across four analytical dimensions: network invariance, global strength invariance, edge invariance, and centrality invariance. Specifically, network invariance and global strength invariance pertain to the comparison of the overall network structure, while edge invariance assesses differences in edge magnitudes, and centrality invariance evaluates centrality measures. The first two components assess the structural equivalence at the level of the entire network, whereas the latter two components respectively evaluate local differences in connection strength and node influence patterns.

## 3. Results

### 3.1. Study Sample

The sociodemographic characteristics of the final sample (n = 1162) are presented in Table 1 in the Participants section. The mean PHQ-9 and NBPS scores were 5.18 (SD = 4.69) and 36.67 (SD = 10.53), respectively. All subsequent network analyses were conducted using the full sample. Table 2 shows the mean scores, standard deviations, strength values, bridge strength values, and predictable values for each symptom of the PHQ-9 and NBPS.

### 3.2. Network Structure and Centrality Measure Analysis

**Figure 1:**
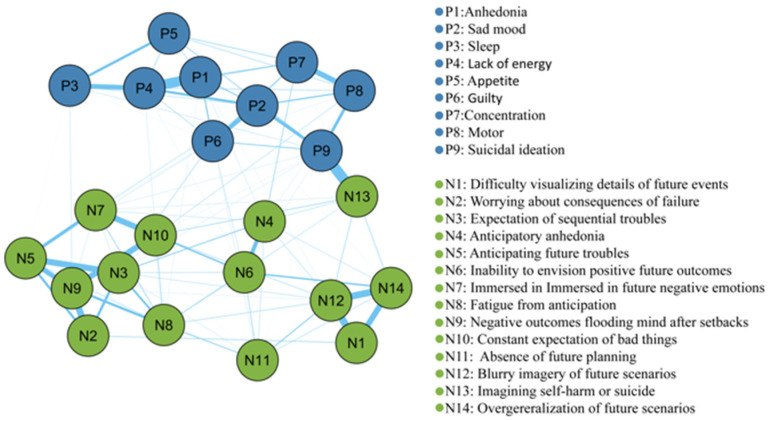
Symptom network model of depression–PB in university students (N = 1162). The blue nodes represent symptoms of depression (PHQ-9), the green nodes represent symptoms of prospection bias (NBPS). The blue line between nodes indicates positive correlation, red indicates negative correlation, and the width of the edge line represents the closeness of the relationship between the nodes.

The network of depression–PB was analyzed using the EBICglasso model (Figure 1). First, 55 edges were not zero (21.74%) among 253 possible edges. Second, the network analysis plot of depression–PB among university students shows that node PHQ1 “anhedonia” and node PHQ4 “lack of energy“ were most closely associated (edge weight = 0.32), followed by the association between node NBPS1 “difficulty visualizing details of future events” and node NBPS12 “blurry imagery of future scenarios” (edge weight = 0.28), node NBPS12 “blurry imagery of future scenarios” and node NBPS14 “overgeneralization of future scenarios” (edge weight = 0.27), node NBPS2 “worrying about consequences of failure” and node NBPS9 “negative outcomes flooding mind after setbacks” (edge weight = 0.27), and node NBPS3 “trouble followed” and node NBPS5 “ruminate on future troubles” (edge weight = 0.26). It is noted that node PHQ9 “suicidal ideation” and node NBPS13 “imagining self-harm or suicide” (edge weight = 0.21) linked depression and negative bias in prospection symptoms.

**Figure 2:**
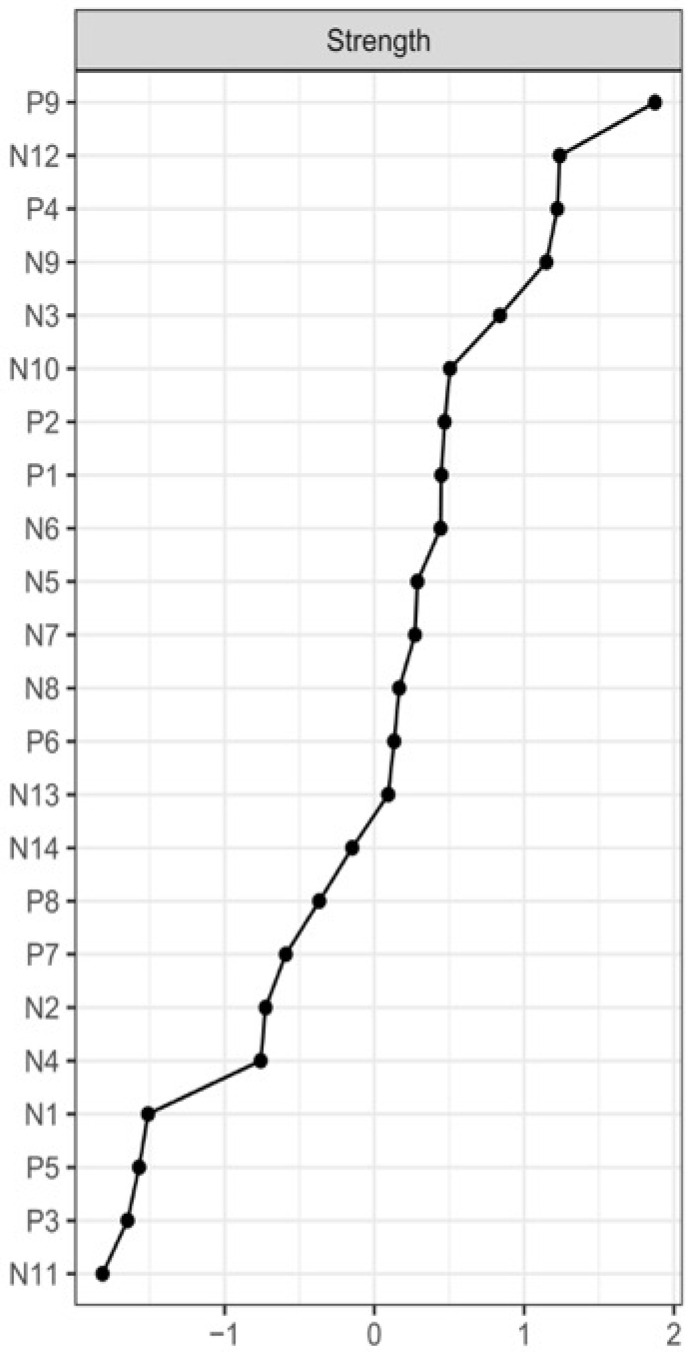
Centrality plot of the node strength in the depression–PB network (N = 1162). Centrality plot depicting the node strength of each symptom in the network. We ranked the value of all symptoms in the network. The strength values decrease from top to bottom.

In order to compare centrality more intuitively, we ranked symptoms in order of value in the plot. Within all symptoms, depression symptoms “suicidal ideation” (strength = 1.34) had the highest strength, followed by NBPS12 (“blurry imagery of future scenarios”, strength = 1.22), PHQ4 (“lack of energy”, strength = 1.22), and NBPS9 (“negative outcomes flooding mind after setbacks”, strength = 1.20; Figure 2). Based on the results of the centrality difference test, the above four symptoms scored higher on the indicator of strength than the other symptoms in the network and can be considered as central symptoms in the symptom network of depression–PB.

The value of node predictability ranged from 0.25 to 0.64, and the average was 0.45. This indicates that on average, 45% of the variance of nodes in the current network can be explained by their neighboring nodes. We ranked predictability value in the depression–PB network. Negative bias in prospection symptom “negative outcomes flooding mind after setbacks” (NBPS9) had the highest predictability (0.64), indicating that 64% of its variance can be explained by its neighbors. Additionally, the depression symptom “appetite” (PHQ5) had the lowest predictability (0.25), indicating that 25% of its variance can be explained by its neighbors (Table 2).

### 3.3. Bridge Symptoms and Bridge Connections

**Figure 3:**
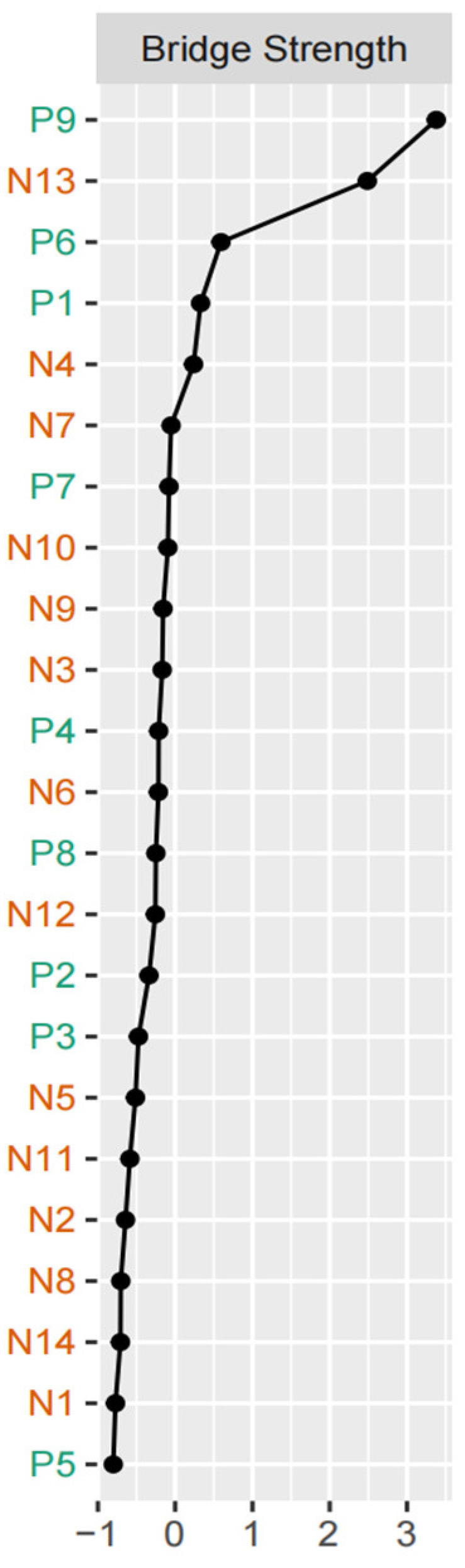
Centrality plot of the bridge strength in the depression–PB network (N = 1162). Centrality plot depicting the bridge strength in the network. The strength values decrease from right to left. Red, NBPS prospection bias nodes; green, PHQ-9 depressive symptom nodes.

In this symptom network, based on the bridging strength of each node, the nodes with a bridging strength greater than the 80th percentile are regarded as bridging nodes (Jones et al., 2021). Therefore, the main bridge symptoms were nodes PHQ9 (“suicidal ideation”, bridge strength = 0.629), NBPS13 (“imagining self-harm or suicide”, bridge strength = 0.496), and PHQ6 (“guilty”, bridge strength = 0.215; Figure 3).

### 3.4. Network Accuracy and Stability

**Figure 4:**
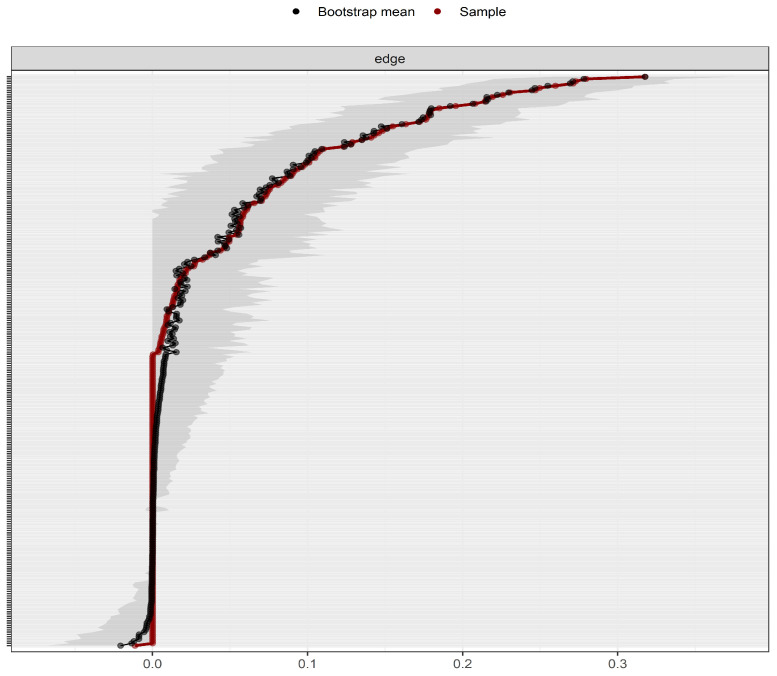
Bootstrap 95% confidence intervals for nonparametric estimation of edge weights. The black dots indicate the value of each edge weight, ordered from the highest to the lowest value. The gray area indicates the 95% confidence interval of the edge weights, estimated using a nonparametric bootstrap procedure (bootstrap net package). Wide intervals indicate low stability, and narrow intervals indicate high stability.

The stability and accuracy of the network structure were preferable in this study. Firstly, a non-parametric estimation method was employed to bootstrap the 95% confidence intervals for the edge weights, revealing that the edge weights are stable (Figure 4). Figure 4 demonstrates the results of multiple edge resampling operations in the network structure. Results suggest good fitting, with most points falling within the confidence interval, indicating relatively stable estimation of network edges. In addition, the stability assessment of nodes within the centrality measures was present in Figure S1. After progressively eliminating samples, the correlation coefficient between the subsample centrality and the original centrality remained above 0.5 (CS coefficient = 0.75), indicating that a maximum sample reduction of 75% could be accepted without significantly affecting the network. Thus, the measurement of network node characteristics was deemed reliable. Finally, the results of the bootstrap difference test for intensity demonstrate that the nodes PHQ4 (“lack of energy”), NBPS3 (“expectation of sequential troubles”), and NBPS9 (“negative outcomes flooding mind after setbacks”) exhibited more significant differences, demonstrating greater concentration compared to other nodes in the network (Figure S2). In contrast, other nodes exhibited milder symptoms, such as NBPS11 (“absence of future planning”), PHQ3 (“sleep”), and NBPS13 (“imagining self-harm or suicide”).

### 3.5. Network Comparison Tests

In this study, we compared the global and local structures between genders (male/female). The results showed no significant differences in network structure (M = 0.18, *p* = 0.431) or global strength (global strength = 10.20 for male students and global strength = 10.00 for female students, S = 0.19, *p* = 0.23) in the network comparison test between male and female students.

## 4. Discussion

The current study aimed at examining the symptom structure of depression and PB among university students using network analysis, with a focus on identifying central and bridge symptoms within the network. The findings indicate that within the depression–PB network, the symptoms of “suicidal ideation” (PHQ9) and “lack of energy” (PHQ4) from the depression domain, along with “blurry imagery of future scenarios” (NBPS12) and “negative outcomes flooding mind after setbacks” (NBPS9) from the PB domain, serve as central symptom nodes. This suggests that these symptoms play a critical role in the activation and maintenance of the depression–PB network. Furthermore, the symptoms of “suicidal ideation” (PHQ9), “imagining self-harm or suicide” (NBPS13), and “guilty” (PHQ6) are bridging symptoms connecting depression and PB.

One of our important findings is that PHQ9 “suicidal ideation” (high strength and bridging centrality) and NBPS13 “imagining self-harm or suicide” (high strength centrality) are core symptoms in the network. This is in line with a meta-analysis that revealed that the standardized centrality index for suicidal ideation in the 15–25 age group was significantly higher than that of other age groups (Robinaugh et al., 2020). Suicidal ideation could lead to an accumulation of negative emotions and social withdrawal, which may subsequently activate other symptoms of depression. The finding that “suicidal ideation” and “imagining self-harm or suicide” as bridging symptoms support Beck’s hopelessness theory, which posits that hopelessness about the future (rather than mere depression) is the strongest predictor of suicidal thoughts (Beck et al., 1985). Previous studies have indicated that suicidal patients reported high levels of suicide-related future imagery and depressive symptoms (Col et al., 2025; Crane et al., 2011).

Suicidal ideation, acting as both a core symptom and a bridge symptom, provides a dynamic interpretative framework for Beck’s cognitive triad theory. Suicidal ideation not only reflects an individual’s negative cognition of the “current self” (Beck et al., 1979), but also forms a cognitive closed loop with future self-destructive simulation through “imagining self-harm or suicide” (NBPS13). The amplification of current reality pain and future hopelessness may be the core driving force and link of the depression–PB symptom clusters. Therefore, these symptoms could serve as important targets for early intervention. Additionally, the low predictability of these two nodes indicates that they may be less influenced by adjacent nodes. It is essential to emphasize direct interventions on these two symptoms in university students, in order to cut the negative loop and prevent the progression of symptoms.

Our results also suggest that “lack of energy” (PHQ4), “blurry imagery of future scenarios” (NBPS12), and “negative outcomes flooding mind after setbacks” (NBPS9) could be central symptoms driving the depression–PB network. This is in line with the previous study indicating that “lack of energy” (PHQ4) was a central symptom in the depression network of university students (Zhao et al., 2023). Lack of energy or fatigue may reinforce the depressive state through behavioral deactivation (Konstantopoulou & Raikou, 2020). This finding supports the “prospection in depression” model, where fatigue is directly related to reduced activity and enhanced negative emotions. These negative real-life experiences influence the formation of PB, further driving the development of depression. On the other hand, NBPS9 and NBPS12 are core manifestations of PB, which stably exist in individuals with depression. Our results also support the CaR–FA–X model of depression (Williams et al., 2007). Negative schemas and rumination could contribute to increased negativity, while functional avoidance and executive function deficits lead to reduced specificity of positive prospections (Ji et al., 2019; Lakshmi et al., 2024).

These three nodes have high predictability, indicating that the influence of adjacent nodes should be considered in future interventions. For example, lack of energy should be intervened jointly with anhedonia and insomnia. Furthermore, comprehensive interventions targeting the specificity, details, and vividness of positive future events should be developed. Moreover, future interventions should block the generation and rumination of negative future events. It is worth noting that previous interventions have mostly focused on improving positive prospection while neglecting the increased negativity. This study suggests that intervening in both increased negativity and reduced positivity is equally important for the early prevention and treatment of depression in university students.

Additionally, the bridge effect of “guilty” (PHQ6) indicates that recurring self-blame could be a psychological mechanism underlying the concurrent depressive and PB symptoms. Therefore, “guilty” may be another potential target for future interventions. This is in line with previous research suggesting that reducing the intensity of self-blame through cognitive modification can enhance the anticipation of future positive events in MDD patients (Amano et al., 2023).

There are some limitations in our study. First, this study is a cross-sectional survey with a relatively small sample size. Future longitudinal studies are needed to verify whether these core symptoms drive dynamic changes in the network. Second, the sample characteristics (e.g., being limited to the Chongqing area and having a higher proportion of females) may limit the generalizability of the results. Future research should also include more comprehensive sociodemographic and health-related information to improve the interpretation and comparability of the findings. Third, we only used self-report measurements, which might induce self-bias and influence the accuracy of the results. Future research should combine objective and subjective assessments to further validate the results. In addition, network analysis examining symptom associations often overlooks the moderating effects of protective factors like resilience and social support, potentially neglecting key targets for intervention design. Finally, given that “suicidal ideation” (PHQ9) and “imagining self-harm or suicide” (NBPS13) emerged as important central and bridge nodes in the present network, future studies should further examine the relationships among depression, PB, and the broader suicide spectrum (suicidal ideation, suicide attempts, and other suicidal behaviors). For example, longitudinal studies including direct measures of suicide attempts are needed to test whether suicide-related future imagery plays a role in the progression from suicidal ideation to suicidal behavior.

## 5. Conclusions

To our best knowledge, the current study is the first to construct a symptom network model integrating depression and PB in university students. This study advances the network theory of psychopathology by demonstrating that suicide-related cognition functions as a critical bridge between present-focused depressive symptoms and future-oriented cognitive distortions. Clinically, the identified central and bridge symptoms may provide useful candidate targets for early identification and intervention among university students. In particular, “suicidal ideation” and “suicide-related future imagery” may help identify students who require closer suicide-risk assessment. These findings may inform the development of more efficient and personalized mental health prevention strategies for university students.

## Figures and Tables

**Table 1:** Demographic characteristics of the study sample (N = 1162).

Variable	Full Sample (n = 1162)	No Depressive Symptoms (PHQ-9 ≤ 4, n = 617)	With Depressive Symptoms (PHQ-9 > 4, n = 545)
Age, mean (SD)	19.47 (1.45)	19.47 (1.43)	19.47 (1.47)
Gender, Female n (%)	709 (61.02%)	390 (63.20%)	319 (58.50%)
PHQ-9 total, mean (SD)	5.18 (4.69)	1.75 (1.47)	9.07 (3.99)
NBPS total, mean (SD)	36.67 (10.53)	32.10 (9.53)	41.85 (9.13)

SD, Standard deviation; PHQ-9, Patient Health Questionnaire-9; NBPS, Negative Bias in Prospection Scale.

**Table 2:** Mean scores, standard deviations, strength, bridge strength, and predictability for each symptom of the PHQ-9 and NBPS.

Variables	M	SD	S	BS	Pre
Depression symptoms (PHQ-9)					
PHQ1: Anhedonia	0.81	0.85	1.07	0.18	0.48
PHQ2: Sad mood	0.58	0.70	1.07	0.08	0.48
PHQ3: Sleep	0.70	0.89	0.66	0.06	0.29
PHQ4: Lack of energy	0.83	0.83	1.22	0.10	0.54
PHQ5: Appetite	0.55	0.81	0.67	0.01	0.25
PHQ6: Guilty	0.52	0.73	1.00	0.22	0.45
PHQ7: Concentration	0.70	0.88	0.86	0.12	0.37
PHQ8: Motor	0.33	0.64	0.91	0.09	0.32
PHQ9: Suicidal ideation	0.17	0.47	1.34	0.63	0.28
Negative bias in prospection symptoms (NBPS)					
NBPS1: Difficulty visualizing details of future events	2.53	1.03	0.68	0.01	0.38
NBPS2: Worrying about consequences of failure	3.15	1.10	0.84	0.03	0.49
NBPS3: Expectation of sequential troubles	2.79	1.09	1.14	0.10	0.61
NBPS4: Anticipatory anhedonia	2.30	1.02	0.83	0.16	0.42
NBPS5: Anticipating future troubles	2.93	1.09	1.03	0.05	0.61
NBPS6: Inability to envision positive future outcomes	2.35	1.03	1.06	0.10	0.53
NBPS7: Immersed in future negative emotions	2.49	1.06	1.03	0.12	0.59
NBPS8: Fatigue from anticipation	2.89	1.18	1.01	0.02	0.58
NBPS9: Negative outcomes flooding mind after setbacks	3.01	1.10	1.20	0.10	0.64
NBPS10: Constant expectation of bad things	2.52	1.02	1.08	0.11	0.59
NBPS11: Absence of future planning	2.73	1.13	0.62	0.04	0.32
NBPS12: Blurry imagery of future scenarios	2.59	1.05	1.22	0.09	0.53
NBPS13: Imagining self-harm or suicide	1.96	1.12	0.10	0.50	0.27
NBPS14: Overgeneralization of future scenarios	2.43	1.12	0.95	0.02	0.46

M, mean; SD, standard deviation; S, strength; BS, bridge strength; Pre, predictability.

## Data Availability

Data are available upon reasonable request. Interested researchers can contact the corresponding authors.

## Supplementary Materials

The following supporting information can be downloaded at: https://www.mdpi.com/article/10.3390/bs16060893/s1, Figure S1: Stability estimations of centrality indices using the case-drop bootstrapping method; Figure S2: Non-parametric bootstrap difference test for strength.

## Abbreviations

The following abbreviations are used in this manuscript:

PB	Prospection bias
SDGs	Sustainable Development Goals
MDD	Major depressive disorder
PHQ-9	Patient Health Questionnaire 9 scale
NBPS	Negative Bias in Prospection Scale
eLASSO	Enhanced least absolute shrinkage and selection operator
EBIC	extended Bayesian Information Criterion
CI	Confidence interval
CS	Correlation stability
NCT	NetworkComparisonTest
SD	Standard deviation
CaR–FA–X	Cognitive Avoidance and Rumination–Functional Avoidance–Executive Function Deficits
FEST	Future event specificity training

## References

[B1-behavsci-16-00893] Amano M., Katayama N., Umeda S., Terasawa Y., Tabuchi H., Kikuchi T., Nakagawa A. (2023). The effect of cognitive behavioral therapy on future thinking in patients with major depressive disorder: A randomized controlled trial. Frontiers in Psychiatry.

[B2-behavsci-16-00893] Beck A. T., Rush A. J., Shaw B. F., Emery G. (1979). Cognitive therapy of depression.

[B3-behavsci-16-00893] Beck A. T., Steer R. A., Kovacs M., Garrison B. (1985). Hopelessness and eventual suicide: A 10-year prospective study of patients hospitalized with suicidal ideation. American Journal of Psychiatry.

[B4-behavsci-16-00893] Borkulo C. V. V., Bork R. V., Boschloo L., Kossakowski J., Tio P., Schoevers R., Waldorp L. (2022). Comparing network structures on three aspects: A permutation test. Psychological Methods.

[B5-behavsci-16-00893] Borsboom D. (2017). A network theory of mental disorders. World Psychiatry.

[B6-behavsci-16-00893] Borsboom D., Cramer A. O. (2013). Network analysis: An integrative approach to the structure of psychopathology. Annual Review of Clinical Psychology.

[B7-behavsci-16-00893] Borsboom D., Robinaugh D. J., Rhemtulla M., Cramer A. O. J. (2018). Robustness and replicability of psychopathology networks. World Psychiatry.

[B8-behavsci-16-00893] Col B. K., Basaran A. G., Kose B. G. (2025). The relationship between e-health literacy, health anxiety, cyberchondria, and death anxiety in university students that study in health related department. Journal of Multidisciplinary Healthcare.

[B9-behavsci-16-00893] Crane C., Shah D., Barnhofer T., Holmes E. A. (2011). Suicidal imagery in a previously depressed community sample. Clinical Psychology & Psychotherapy.

[B10-behavsci-16-00893] Epskamp S., Borsboom D., Fried E. I. (2018). Estimating psychological networks and their accuracy: A tutorial paper. Behavior Research Methods.

[B11-behavsci-16-00893] Fischer E., Glashauser A., Laireiter A. R., Fave A. D. (2022). Development and evaluation of a prospective group coaching program: Increasing well-being and openness to the future in a subclinical sample. Journal of Happiness Studies.

[B12-behavsci-16-00893] Foygel R., Drton M. (2010). Extended Bayesian information criteria for Gaussian graphical models. Advances in Neural Information Processing Systems.

[B13-behavsci-16-00893] Friedman J., Hastie T., Tibshirani R. (2008). Sparse inverse covariance estimation with the graphical lasso. Biostatistics.

[B14-behavsci-16-00893] Fu X., Zhang K., Chen X., Chen Z. (2022). Report on national mental health development in China (2021–2022).

[B15-behavsci-16-00893] Hallford D. J. (2019). The phenomenological characteristics of autobiographical future thinking in dysphoric and non-dysphoric individuals. Psychiatry Research.

[B16-behavsci-16-00893] Hallford D. J., Rusanov D., Yeow J. J. E., Austin D. W., D’Argembeau A., Fuller-Tyszkiewicz M., Raes F. (2022). Reducing anhedonia in major depressive disorder with future event specificity training (FEST): A randomized controlled trial. Cognitive Therapy and Research.

[B17-behavsci-16-00893] Haslbeck J. M. B., Waldorp L. J. (2018). How well do network models predict observations? On the importance of predictability in network models. Behavior Research Methods.

[B18-behavsci-16-00893] Herrman H., Patel V., Kieling C., Berk M., Buchweitz C., Cuijpers P., Wolpert M. (2022). Time for united action on depression: A Lancet-World Psychiatric Association Commission. Lancet.

[B19-behavsci-16-00893] Holmes E. A., Crane C., Fennell M. J., Williams J. M. (2007). Imagery about suicide in depression—“Flash-forwards”?. Journal of Behavior Therapy and Experimental Psychiatry.

[B20-behavsci-16-00893] Ji J. L., Holmes E. A., Macleod C., Murphy F. C. (2019). Spontaneous cognition in dysphoria: Reduced positive bias in imagining the future. Psychological Research.

[B21-behavsci-16-00893] Jones P. J., Ma R., McNally R. J. (2021). Bridge centrality: A network approach to understanding comorbidity. Multivariate Behavioral Research.

[B22-behavsci-16-00893] Konstantopoulou G., Raikou N. (2020). Clinical evaluation of depression in university students during quarantine due to COVID-19 pandemic. European Journal of Public Health Studies.

[B23-behavsci-16-00893] Kube T., Schwarting R. K. W., Rozenkrantz L., Glombiewski J. A., Rief W. (2020). Distorted cognitive processes in major depression: A predictive processing perspective. Biological Psychiatry.

[B24-behavsci-16-00893] Lakshmi P. M., Kishore M. T., Roopesh B. N., Jacob P., Rusanov D., Hallford D. J. (2024). Future thinking and anticipatory pleasure in adolescents with major depression: Association with depression symptoms and executive functions. Clinical Child Psychology and Psychiatry.

[B25-behavsci-16-00893] Lin Q., Bonkano O., Wu K., Liu Q., Ali Ibrahim T., Liu L. (2021). The value of Chinese version GAD-7 and PHQ-9 to screen anxiety and depression in Chinese outpatients with atypical chest pain. Therapeutics and Clinical Risk Management.

[B26-behavsci-16-00893] Lin Z. Z., Cai H. W., Huang Y. F., Zhou L. L., Yuan Z. Y., He L. P., Li J. (2025). Prevalence of depression among university students in China: A systematic review and meta-analysis. BMC Psychology.

[B27-behavsci-16-00893] Namaky N., Glenn J. J., Eberle J. W., Teachman B. A. (2021). Adapting cognitive bias modification to train healthy prospection. Behaviour Research and Therapy.

[B28-behavsci-16-00893] Richardson L. P., Mccauley E., Grossman D. C., Mccarty C. A., Katon W. (2010). Evaluation of the patient health questionnaire-9 item for detecting major depression among adolescents. Pediatrics.

[B29-behavsci-16-00893] Robinaugh D. J., Hoekstra R. H. A., Toner E. R., Borsboom D. (2020). The network approach to psychopathology: A review of the literature 2008–2018 and an agenda for future research. Psychological Medicine.

[B30-behavsci-16-00893] Roepke A. M., Seligman M. E. (2016). Depression and prospection. British Journal of Clinical Psychology.

[B31-behavsci-16-00893] Tang P., Pavlopoulou G., Kostyrka-Allchorne K., Phillips-Owen J., Sonuga-Barke E. (2023). Links between mental health problems and future thinking from the perspective of adolescents with experience of depression and anxiety: A qualitative study. Child and Adolescent Psychiatry and Mental Health.

[B32-behavsci-16-00893] Williams J. M. G., Barnhofer T., Crane C., Herman D., Raes F., Watkins E., Dalgleish T. (2007). Autobiographical memory specificity and emotional disorder. Psychological Bulletin.

[B33-behavsci-16-00893] Yang Z. Y., Zheng Y. C., Yang X., Wang Y. T., Feng Z. Z. (2023). The development of the Negative Bias in Prospection Scale: A novel assessment of dysfunctional prospection in depression. Psych Journal.

[B34-behavsci-16-00893] Zhao Y., Qu D., Chen S., Chi X. (2023). Network analysis of internet addiction and depression among Chinese university students during the COVID-19 pandemic: A longitudinal study. Computers in Human Behavior.

