# Supplementary material for: A Network Analysis of Suicidal Cognition as a Central Node Linking Depression and Prospection Bias in University Students
Source: Behav Sci (Basel). 2026 Jun 2;16(6):893. doi: 10.3390/bs16060893 (PMC13296312; doi:10.3390/bs16060893)
Supplement: Supplementary file 1 [file behavsci-16-00893-s001.zip › behavsci-4240038-supplementary.pdf]

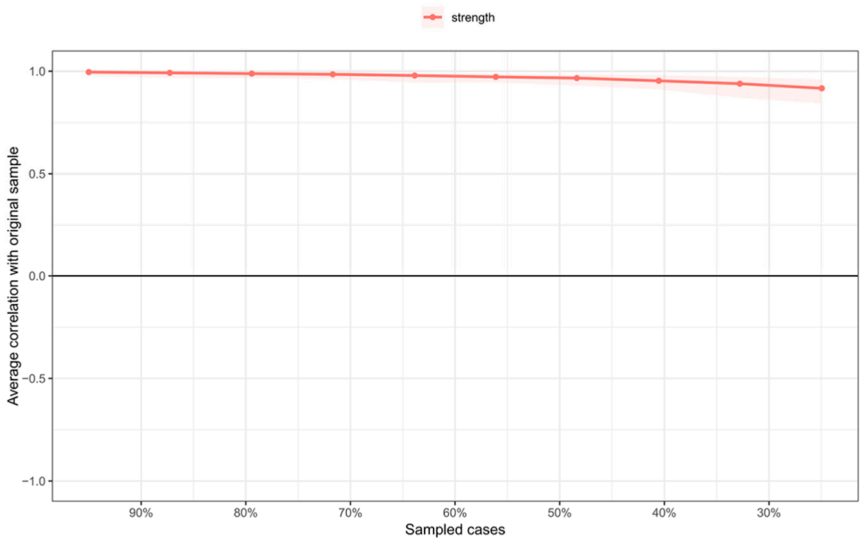

Figure S1. Stability estimations of centrality indices using the case-drop bootstrapping method (N=1162). X-axis is the percentage of samples used at each step, and y-axis is the degree of association between the original sample and the estimated results from the sample reduction. The red line indicates the specific value of the node in emphasizing centrality.

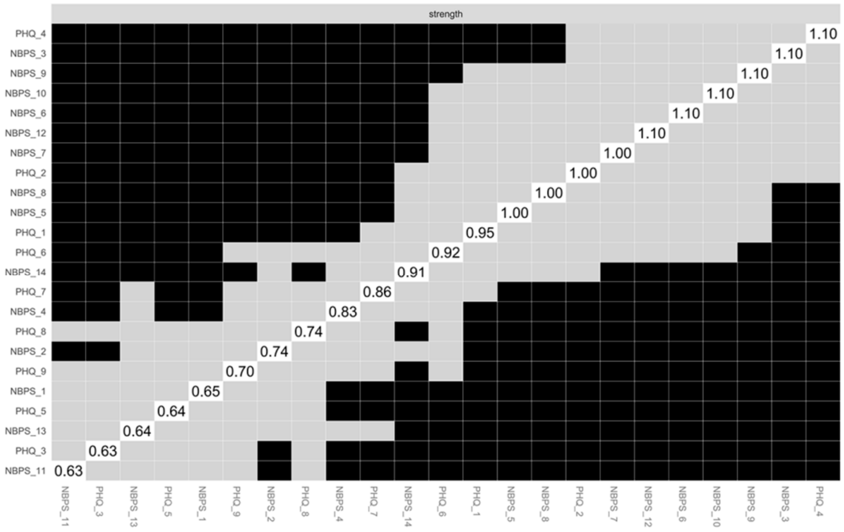

Figure S2. Non-parametric bootstrap difference test for strength. Gray boxes indicate non-significant differences between nodes, while black boxes indicate significant differences (α =0.05). The values reported in the diagonal line indicate the strength values of the nodes.
